# A Decentralized Marketplace for Patient-Generated Health Data: Design Science Approach

**DOI:** 10.2196/42743

**Published:** 2023-02-27

**Authors:** Hemang Subramanian

**Affiliations:** 1 Department of Information Systems and Business Analytics College of Business Florida International University Miami, FL United States

**Keywords:** decentralized marketplace, smart contracts, nonfungible tokens, patient-generated health data

## Abstract

**Background:**

Wearable devices have limited ability to store and process such data. Currently, individual users or data aggregators are unable to monetize or contribute such data to wider analytics use cases. When combined with clinical health data, such data can improve the predictive power of data-driven analytics and can proffer many benefits to improve the quality of care. We propose and provide a marketplace mechanism to make these data available while benefiting data providers.

**Objective:**

We aimed to propose the concept of a decentralized marketplace for patient-generated health data that can improve provenance, data accuracy, security, and privacy. Using a proof-of-concept prototype with an interplanetary file system (IPFS) and Ethereum smart contracts, we aimed to demonstrate decentralized marketplace functionality with the blockchain. We also aimed to illustrate and demonstrate the benefits of such a marketplace.

**Methods:**

We used a design science research methodology to define and prototype our decentralized marketplace and used the Ethereum blockchain, solidity smart-contract programming language, the web3.js library, and node.js with the MetaMask application to prototype our system.

**Results:**

We designed and implemented a prototype of a decentralized health care marketplace catering to health data. We used an IPFS to store data, provide an encryption scheme for the data, and provide smart contracts to communicate with users on the Ethereum blockchain. We met the design goals we set out to accomplish in this study.

**Conclusions:**

A decentralized marketplace for trading patient-generated health data can be created using smart-contract technology and IPFS-based data storage. Such a marketplace can improve quality, availability, and provenance and satisfy data privacy, access, auditability, and security needs for such data when compared with centralized systems.

## Introduction

### Background

Pervasive devices and wearables create health data that can be combined with electronic health record data to improve disease predictability. Such data can be used to create a patient-centric health system in addition to managing population health [1,2]. There are limited examples of patient-generated health data (PGHD) in clinical settings; however, recent advances in predictive analytics and health informatics have found numerous uses for such data. For example, mobile data may be used to predict and provide early warning signs of diseases such as hypertension, diabetes, cancer, and other heart ailments [3]. PGHD assets can become important value-adding differentiators for health care–related businesses, adding value across the health care value chain [4]. However, the design of centralized warehouses to support clinical and translational research suffers from many challenges, including “organization of data,” “access control,” “oversight and governance,” “sharing of data,” “service management between different bodies such as informatics and bio-statisticians,” and “technology challenges of maintenance, upgradation, and storage” [5]. In addition, Kruse and Goswamy [1] describe various challenges with data structure organization, validation, security, and privacy. PGHD available for real-time analysis may be challenging because device manufacturers often control all data supply, or data are often deleted because edge devices (mobile and pervasive) are not designed to include long-term memory storage [6].

Mainstream clinical health care repositories, such as a research patient data repository (RPDR) and health information exchanges (HIEs), are examples of large complex data warehouses often governed by consortiums. RPDRs specify rules for data collection and access among members, which are focused on the clinical data field [4,5]. In the RPDR, health care data storage and analysis are distributed among consortium members, with specific well-vetted guidelines for data access. Gagalova and Elizalde [5] describe the creation of an integrated data repository with the following steps: data extraction, deidentification, ID assignment, transformation, ontology mapping, linkage, and loading into warehouses, among the stages for data retrieval. Recent innovations in web service–based application programming interfaces (APIs) and the evolution of standards have provided standards such as Fast Healthcare Interoperability Resources, which enable third-party systems to access clinical health care data [2]. However, these mechanisms depend on the ability of independent data stores, hospital systems, and data intermediaries to satisfy legal mandates. Access mechanisms cannot be applied to patient-generated data where data are stored by device manufacturers or third-party vendors [6].

Preaggregated anonymized health data sets are available for sale and subscription through Amazon Web Services such as Qiagen [7], IBM Watson [8], Medisafe [9], and Annotate-it [10]. Such data can be used for analysis in several domains, such as cardiology or pathology, to discover and predict diseases using sophisticated machine learning models. Centralized data stores, such as research data repository and HIEs, are alternatives, but hospital systems usually store clinical data, not PGHD [3]. In addition, PGHD data sets need not provide the necessary provenance (eg, one cannot request the source or transmission records for data because they are subscription-based). Similarly, it would be difficult to verify the recency of such data because they are already curated from publicly available information or by the firm offering subscription-based services. Prior research has recommended standardizing formats for data storage to exchange health care data (such as the Health Level Seven [international standards for transfer of clinical and administrative health data]) and to create APIs such as Fast Healthcare Interoperability Resources that can seamlessly operate across clinical systems; accomplishing such a standard would need legal mandates [11].

This paper proposes, designs, and provides a proof-of-concept implementation for a secure public blockchain infrastructure–based PGHD marketplace that can address several issues concerning data reliability, privacy, provenance, and availability. In this paper, we proposed a user-level encryption schema that enables a seamless exchange and monetization of health data by creators. Users are incentivized to produce high-quality data sets on the supply side of such a marketplace. On the demand side, users experience reduced search costs and can locate and trade with high-quality data providers at a lower price because of competition and choice. In this study, we examined the following research questions:

- Research question 1: How can we design a decentralized PGHD marketplace?
- Research question 2: What are the benefits of decentralized data?

We argue that a marketplace approach can be a panacea for many health data–quality concerns and issues through (1) market-induced competition in a decentralized marketplace resulting in increased availability, (2) backed by privacy and an encryption schema that protects data provider privacy and ownership, and (3) a reputation mechanism for data sets and market participants, while (4) simultaneously enabling monetary incentives for participants, including the infrastructure provider or marketplace creators. Next, we examined data storage and access challenges.

### The Need for a PGHD Marketplace

#### Overview

In a health data marketplace, different sellers, buyers, and (value-added) service providers congregate to cocreate value for the entire ecosystem [12]. Users who own health care record data can assign agents to operate on their behalf or directly benefit economically by having the ability to sell such data [13]. Data aggregators, health care data repository owners, or storage providers can monetize health data by enabling value-added services, such as applying intelligent data analytics and prescriptive or diagnostic machine learning technologies to their data [14]. A PGHD marketplace has to adhere to the legal requirements of privacy and data access [6]. However, substantial private trade in health care technology, curated data sets, and secondary uses of such data sets have existed for a time. Private entities with resources, that is, both human resources and financial and technical know-how, have been able to arbitrage the advantages of such PGHD data sets by solving unique predictive problems.

On the one hand, technology has enabled autonomous driving with high accuracy [15]; on the other hand, it is not yet possible for automated disease diagnosis or prediction without specialist intervention from data. The lack of automated diagnosis from PGHD data increases the costs of diagnosis, not to mention delays in diagnosis [10]. In addition, such asymmetrical market power between resourceful players and smaller health care analytics startups can reduce the discovery time for newer data-driven models for diagnosis [16]. Often, health data sets are expensive and do not provide any value to creators. For example, the health data set for predicting heart disease costs US $500 per hour for use on Amazon Sage Maker.

On the seller’s side, data providers, aggregators, or intermediaries cannot monetize the precious data created. Another issue is that of provenance, where it is not possible for the analyst or others to truly validate or ascertain, under confidentiality, the creator of such data. Similarly, on the buyer’s side, small- and medium-scale businesses and research projects that need large data sets to perform experimental analysis face an entry barrier because of the lack of data provenance [16]. Clinical studies are backed by stringent data disclosure and ethics reviews, where such reviews provide value in preventing data fabrication and unethical uses of data. Applying similar stringent data disclosure standards to collect and access PGHD may be possible if a marketplace approach is used, wherein users are compensated for sharing their own data [17], and moderation mechanisms filter out fabricated data. In many fields of medicine and health care, such as digital pathology, the lack of a large corpus of data for training algorithms in image detection and pattern analysis, owing to lack of data, is challenging. However, recent improvements in using patient health data are visible in research done by Google Inc [18] and Apple Inc [19]. The lack of automation increases the cost of care and, in many cases, prevents improvements to health care that are technically feasible yet lack data accessibility, data provenance, and data quality [20-22]. Next, we discuss the key properties of a PGHD marketplace.

#### Properties of a Decentralized PGHD Marketplace

The unique properties of a PGHD marketplace include its ability to preserve data privacy, access control, data storage, and fault tolerance. Buyers who purchase and use such data to develop useful classification algorithms monetize the data. In addition, such analytics enable various auxiliaries, such as analytics for diagnoses, disease prediction, and gamification of health care services [23]. Blockchains are a new distributed and decentralized technology used to address the challenges of data standardization, system interoperability, security, privacy, and accessibility [24]. Before the advent of blockchains, providing anonymized, privacy-controlled single points of access for different data sources for each user was a challenging problem [25]. We present the design and implementation of a decentralized blockchain-based marketplace. A decentralized marketplace enables faster matching of buyers and sellers of data, seamless transaction efficiency, and institutional infrastructure features, such as provenance, privacy, access control, and perennial storage [12].

#### Scope of the Marketplace

**Figure 1:**
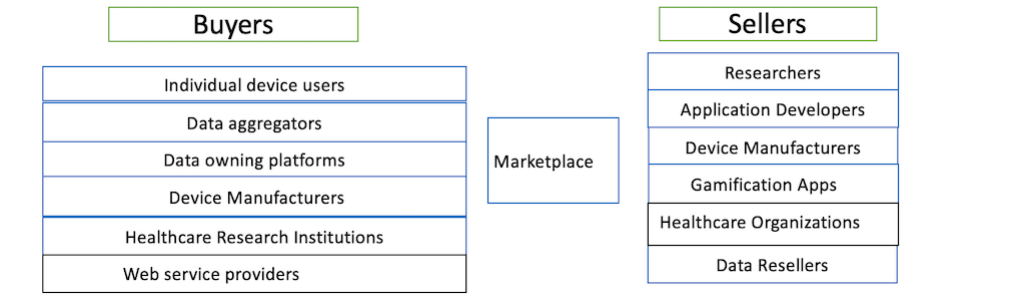
Decentralized health care data marketplace.

Figure 1 describes the 2 sides of such a marketplace and the actors in the marketplace.

Marketplaces are 2-sided, with buyers on one side and sellers on the other. Buyers can purchase data to modify, analyze, and sell downstream or use it for research and other purposes. The buyer side consists of service providers, such as data aggregators, individual patients who can share personal health care data, firms that provide predictive analytics for data, and application developers or researchers or data scientists who analyze data and add value. The buyer side could also consist of specialists who resell data, data aggregators, game developers, and research institutions. The scope of the data seller entails only PGHD, wherein the patient is responsible for creating such data using personal devices. Others, such as health research institutions, web service providers, and data aggregators, form a part of the supply chain wherein the patient authorizes them to intervene. Table S1 in Multimedia Appendix 1 describes the differences between centralized health data stores and decentralized PGHD data marketplaces targeted in our design.

The burden of the cost of data storage for centralized and managed health information systems such as the RPDR or HIEs usually falls on the patient or the end user [23]. A marketplace is not feasible in such data architectures because HIEs specifically cater to clinical health care data not PGHD data. Table S2 in Multimedia Appendix 1 describes the differences between decentralized PGHD data stores and HIEs and integrated data repositories.

Centralized data stores often do not cater to PGHD, which can come from either the patient’s own health device or from another device, such as a publicly available blood pressure monitor, commonly found in grocery stores. However, very often, such data can provide valuable insights into user health and when services are aggregated into apps, such as the one by Google [18] or by Sleep Tracker [26].

Blockchains have been shown to provide various benefits when user data are involved, allowing users to store large quantities of data [6]. However, such benefits are not transferred to pervasive devices and ubiquitous applications that are designed with security, access, privacy, and performance considerations. Prior health care research on data at health care exchanges, tamper-proofing data, and securing data has demonstrated benefits in the context of health care [21]. In the subsequent section, we discuss the data-quality dimensions pertaining to health care data and how a decentralized marketplace addresses quality issues. There are three main dimensions to data quality in decentralized marketplaces: (1) information quality, (2) security, and (3) communication.

Information quality refers to the following 7 characteristics:

- Usability: the more usable the data, the more buyers there are for such data. Owing to the digital nature of the data set, data can be replicated easily and sold to downstream users either as is or by adding other features and analytics, such as tags.
- Timeliness: sellers must ensure the timeliness of data that are submitted for sale on the market. Otherwise, they will lose out on more current data. In addition, blockchains record and timestamp every record that has been uploaded, preventing users from altering the actual event in the data set.
- Relevance: sellers will only share or upload relevant data for sale in the marketplace. Irrelevant or falsified data records will be penalized by other users and can affect user reputation in the marketplace.
- Consistency: data will have to be consistent by ensuring that similar data formats are used. Marketplace operators can enforce templates or buyers can solicit data in particular formats. Consequently, users will only upload consistent data to the marketplace.
- Completeness: the data will have to be complete, and users can seek panel data. Data providers will be either individual users or intermediaries.
- Accuracy: the data can be checked for accuracy on the blockchain because transaction rules can be written into smart contracts. Basic syntax checking and advanced analytics-based checks can be conducted on these data.
- Access rights management: user- or firm-based encryption at the wallet level encrypts data. Similarly, only those with private keys will have access to it.

Security refers to the following 4 characteristics:

- Privacy: the blockchain, by design, ensures that data are accessible by only those who possess the keys (refer to Key Management in Multimedia Appendix 2). In addition, an encryption mechanism implemented by the module makes the data inaccessible to other users. Privacy can also be ensured by preventing deanonymization [27] of data by fixing access through encryption keys and by allowing only the data owners access.
- Confidentiality: the decentralized marketplace app can encrypt or decrypt the data with the user’s private key to ensure the confidentiality of data to only those who possess access.
- Secure access: access to data is plausible only through a secure private-public key maintained by the user. Data encrypted by a user can only be unencrypted through the platform or when the user provides the buyer with a key offline.
- Governance of data: the marketplace can ensure a governance model, such as a consortium-based decentralized autonomous organization, is responsible for all major governance decisions. Such mechanisms have been used in large public blockchain projects, such as the maker foundation.

Data communication refers to the following 3 characteristics:

- Provenance: the digital transaction records on the blockchain will enable data to be traced back to the source on the blockchain.
- Interpretability: the different people in the network chain must interpret the data in a similar manner through a protocol.
- Transmissibility: the data are transmissible to other owners using the blockchain.

## Methods

### Overview

We used the design science research methodology [28,29], commonly used in information systems and computer science, to design and validate the decentralized marketplace.

### Design Science Research Methodology and Our Approach to the Solution

#### Overview

The following are 3 phases in the design science research method:

- Phase 1: the discovery phase consists of (1) the problem definition and the importance of the problem and (2) identification of the objectives of the solution.
- Phase 2: the solution implementation phase consists of (1) prototype design and development and (2) demonstration.
- Phase 3: the evaluation phase consists of (1) an evaluation of the artifact against requirements and (2) a discussion of the results and implications.

#### Phase 1: Problem Definition and Importance of Solving

Beinke and Fitte [28] discuss that blockchain technology offers the possibility to verify transactions through a decentralized network and identified 34 stakeholder-specific requirements. Although their proposed blockchain-based architecture caters to electronic health records, certain requirements to support PGHD marketplaces are extracted and summarized in the following goals, along with the justification in the subsequent section.

The design goals of PGHD marketplace are as follows:

- Goal 1: data access—data access must be allowed between different sellers and buyers, that is, buyers must have the right to access the data they purchased and sellers should have the ability to own and control the data and copies of data
  Justification: buyers and sellers can create and hold data only they can access. This creates a marketplace where true ownership is recorded on the blockchain and is verifiable by anyone.
- Goal 2: marketplace workflow functionality—marketplace functionality for data should be enabled, that is, sellers should offer a pricing mechanism (either fixed or auction-like) to trade data, and buyers should be able to purchase the same with cryptocurrencies
  Justification: data should have value (a floor price) in the system, and the data owner (creator) should access the value. Those who purchase data can pay for the data on the platform. Similarly, payment and transfer of the service are fully automated and do not need an intermediary to validate and transfer transactions.
- Goal 3: data encryption support—support for data encryption to ensure that only the owner can access the data and other users cannot see the data
  Justification: the data encryption schema for publicly stored information is a critical component for maintaining data privacy.
- Goal 4: Health Insurance Portability and Accountability Act (HIPAA) compliance—support for HIPAA compliance by providing users with information privacy and the ability to purge data from the marketplace offering
  Justification: the system must adhere to different criteria laid out by HIPAA-compliant mobile use.
- Goal 5: reputation model—support for reputation model and data fabrication defenses
  Justification: the validity of the data provider (patient) or patient representative must be checked in the marketplace. Similarly, data fabrication cases must be penalized upon detection.

#### Phase 2: Design and Implementation

We proposed an approach using nonfungible token (NFT) standards (Ethereum Request for Comments [ERC]-721 and ERC-1155) optimized for PGHD data and propose the creation of decentralized health care marketplaces where there are sellers, buyers, and value-added service providers, among others (Figure 1). Each participant in the marketplace, that is, seller, buyer, or value-added service provider, is identified by their wallet addresses (a modified version of their public key on the blockchain) [30]. Marketplace participants adhere to privacy, data security, and other features required by laws, such as HIPAA and General Data Protection Regulation. Subramanian and Subramanian [31] described a digital pathology system using an interplanetary file system (IPFS) and Ethereum. We used a similar strategy for our design, except that we built a full marketplace based on smart contracts with user encryption of data, the IPFS to store the data, and the web3 interface to enable interactions between buyers and sellers. We reduced the transaction fees needed to operate a public blockchain infrastructure to a few cents on Ethereum version 2 (proof of stake) [28].

### A Decentralized PGHD Data Marketplace Using Smart Contracts Using NFT Standards, IPFS, and MongoDB

The subsequent section is an overview of key technologies used to create our decentralized marketplace, based on NFTs. First, we examined how the blockchain network enables a decentralized marketplace. Then, we studied the principles of Ethereum-based smart contracts. Finally, we analyzed how decentralized markets powered by Ethereum-based smart contracts enable NFT markets to make them function. The Ethereum blockchain enables a wide range of transactions via smart contracts and self-executable Turing-complete programs, which run on the Ethereum virtual machine and maintain a state in their storage. The Ethereum virtual machine has a stack-based architecture and can store things on the stack (eg, using bytecode operations), in memory (eg, temporary variables within functions), or in storage (eg, permanent variables holding database entries). Each smart contract can read and write data only to its smart-data structure. The network consensus mechanism determines which user in the network will append the transactions to the chain as a new block. Ethereum has recently moved to the proof of stake mechanism, which substantially reduces energy consumption [32]. With proof of stake, a network algorithm determines which node will add the block to the chain based on the node’s stake, a combination of parameters, including their account balance. The transaction fee for smart-contract operations, such as minting, transferring data, and creating an on-chain record, is a fraction of a cent on Ethereum proof of stake.

### PGHD as NFTs Listed in Marketplaces

Smart contracts provide an opportunity to develop applications with complex functionalities in a blockchain network. Using Ethereum smart contracts, we implemented the ERC-721 standard with which we can store, mint, list, trade, and burn health care data. We also implemented recurring revenue for data creators and owners and facilitated the provision of quality-of-service paradigms for the market. The life cycle of an NFT is presented in a list here in the context of the tokens on the network. The details of each stage are provided:

- Storage: the data are uploaded onto IPFS and are kept there for storage.
- Mint: the content identifier (CID) returned by IPFS is returned to the blockchain. The token is minted.
- List: the minted token is used to list the data set for other users to purchase on the blockchain. This listing will use MongoDB to store and retrieve for the user interface and list the data on the blockchain.
- Trade: users who purchase the token will be able to do so using their crypto token balances from their wallets. Then, they will be able to transfer the token ID’s ownership (and hence, data access) to themselves. Such data access will be recorded on the blockchain.
- Burn: finally, the token, based on the owner’s choice, can be burned, and access can be removed from the actual data set by transferring the token to a NULL address.

**Figure 2:**
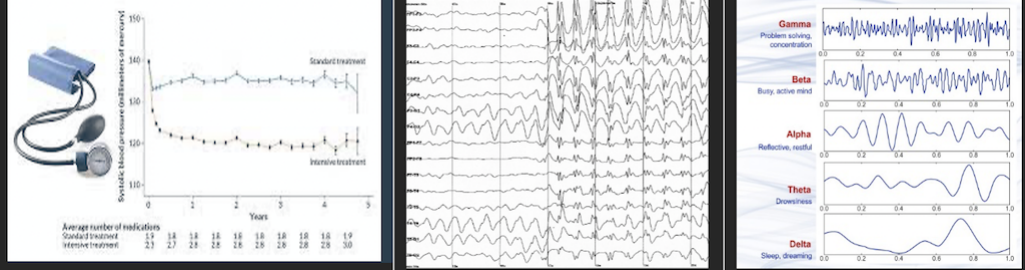
Blood pressure data, electroencephalograph, and brainwave data pertaining to a patient collected on her own personal devices.

Figure 2 depicts the different variations of data stored on the blockchain. The metadata separates the ownership of data from the user uploading the data to the marketplace. Buyers of these data can use it to analyze and provide value-added services to end users of the marketplace. They can also reupload data to the marketplace or relist data as is. The marketplace provides financial incentives to data creators and marketplace-hosting agencies to ensure that the system works per design. Similarly, each time a data owner uploads data, they can claim a royalty on each future sale. Similarly, the marketplace wallet can receive a fixed amount of cryptocurrency as a commission per sale, making it financially feasible to maintain future requirements for the platform.

**Figure 3:**
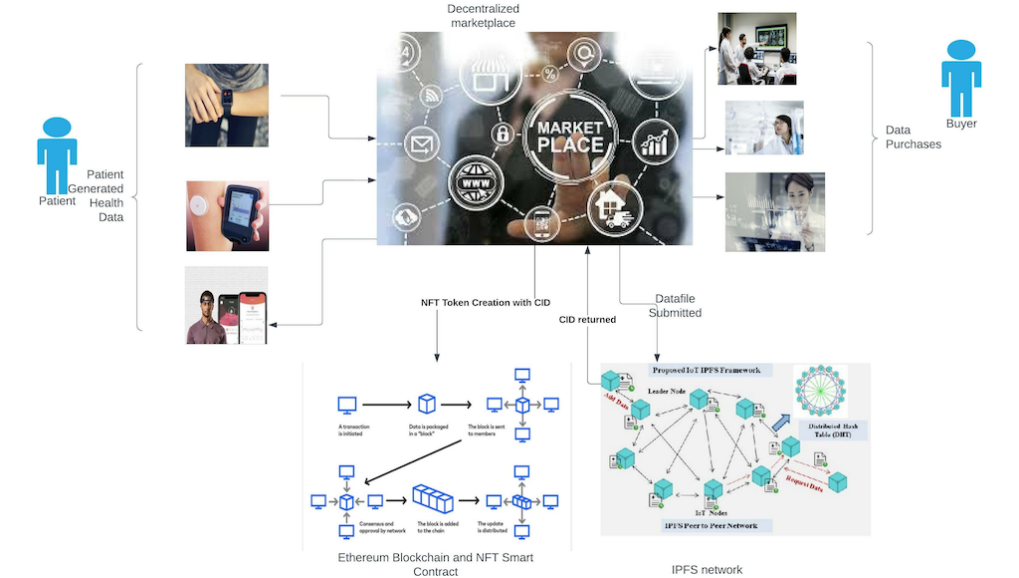
This diagram shows the transactions among data creators, buyers, the interplanetary file system (IPFS), and the blockchain. CID: content identifier; NFT: nonfungible token.

Sellers can set prices for the data sets listed, and once a sale transaction occurs, the cryptocurrency will be transferred to the seller after deducting platform fees and royalty fees preset in the smart contract. The architecture of such a marketplace is illustrated in Figure 3. The PGHD data are stored in the IPFS, and the data identifier CID is stored on Ethereum within a smart contract (ERC-721). The marketplace connects the data creators and the buyers through the IPFS and Ethereum infrastructure. The data are encrypted on the IPFS as per the protocol discussed in the multiparty and encryption schema [32] discussed in subsequent sections.

The PGHD will be stored on the IPFS, and the corresponding token ID will contain the metadata associated with the data owner. Similarly, each time the record or the token changes hands, the token will be transferred to a new owner, and the new owner will access the data. In between the data transfer, the encryption protocol is invoked, which generates a new pair of keys and provides the new owner with the key to decrypt the data. Consequently, the blockchain records the owner of the data, which in turn points to the CID on the IPFS. The marketplace creator can use a database, such as MongoDB, to store the mappings of user wallets, CIDs of data, and corresponding price variables, as in our case. This database is not absolutely essential but can be used to supplement data stored on the blockchain for faster lookup and querying or searching of data to provide ease of use to the user.

Users can upload multiple copies of their data to the IPFS. Each copy of the data must go through the minting workflow. In the minting workflow, data are newly uploaded onto the IPFS and encrypted with a different key. Later, this new IPFS CID will be minted as a separate token for listing. The platform does not restrict offering multiple data sets belonging to the same user. However, marketplace moderation mechanisms can flag duplicates uploaded onto the system or can potentially affect the reputation of the user.

**Figure 4:**
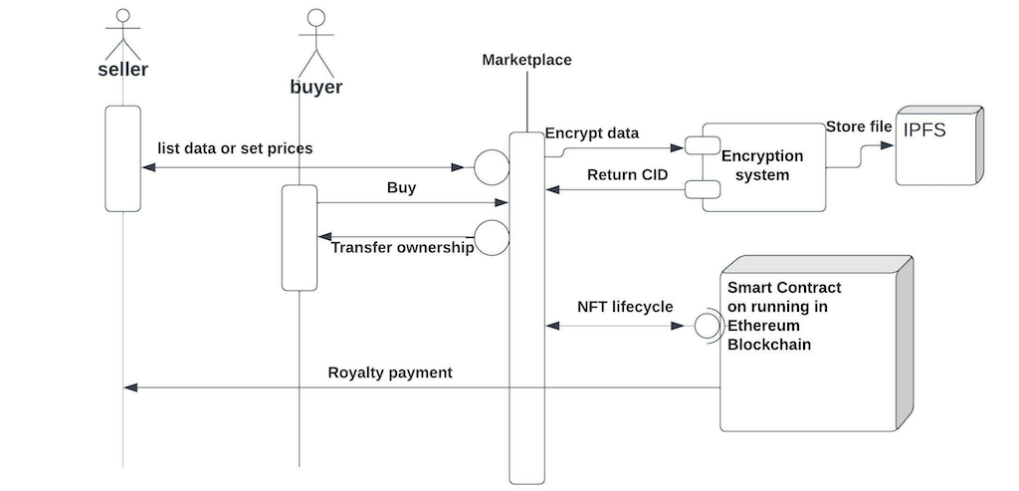
Design schematic and architecture of a decentralized marketplace prototype. CID: Content Identifier; IPFS: interplanetary file system; NFT: nonfungible token.

In Figure 4, we list the schematic and user flow of such a PGHD marketplace. Creators of data or owners of digital data, for example, patients and hospital systems, can list their data on the marketplace using an easy-to-use user interface. Sellers are identified on the blockchain through a know-your-customer and antimoney laundering mechanism as well as their wallet addresses associated with their purchases. A preview image illustrates the sample data sets used. The actual data set forms a part of a JSON text entry. The data are stored on the IPFS, a distributed file system hosting peer-to-peer file storage. If the public IPFS is not sufficiently performant, marketplace creators can use layer 2 solutions, such as Filecoin, ArWeave, and Storj. As data scale to petabytes or exabytes, a layer 2 solution will be required because the IPFS may not be performant enough in terms of response times for the download of data unless the marketplace provides its own hosting and pinning service.

Similarly, the buyers of data purchase the data from the owner. In the process, the NFT’s ownership is transferred to the buyer, which is recorded on the blockchain. In addition, we have third-party data validators and analysts such as “value-added service” providers who will purchase the data from the marketplace, perform operations such as data-oriented simulations, data mining, or cleaning of data and relist them or resell them downstream.

### Health Data Encryption

#### Overview

**Figure 5:**
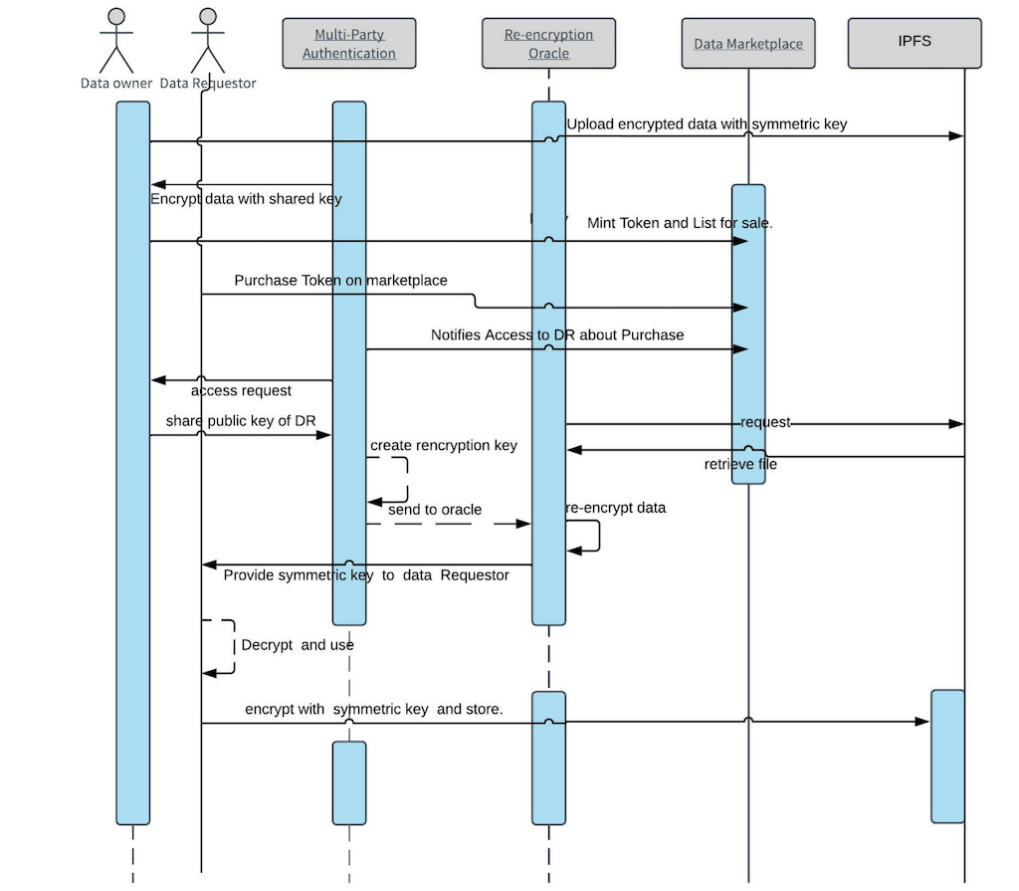
Activity diagram for data encryption flow in the data marketplace with buyer and seller. DR: data requestor; IPFS: interplanetary file system.

O’Donoghue et al [33] discussed various trade-offs to be managed adaptively to improve electronic medical record utility and argued that although these trade-offs can result in improved blockchain security, some of these features could affect scalability. Kumar and Bharti [34] summarized 10 different approaches for encrypting IPFS data records using various encryption methods and described different storage solutions. In addition, a recent work by Lin and Zhang [35] proposed an approach to create a directory-based file system and to use the bit swap protocol built on the IPFS to transfer encrypted records among users. As a technology, we could apply any of the 10 encryption approaches. We chose a modified version of the multiparty authentication and re-encryption oracle suggested by Battah et al [32], who released their full code. In brief, the activity diagram for the encryption schema is shown in Figure 5. The main entities in the multisignature system are multiparty authentication servers, the re-encryption oracle, the data owner, and the data requester.

The data owner (seller) uploads the data and agrees with access requirements posed by the multiparty authenticator or multiparty authentication server. The data owner registers the address of the data (which is the hash of the data) on the blockchain by minting the token once the multiparty authentication server and encryption oracle encrypt the data. There is always a shared wallet between the multiparty authenticator and the data owner on the system, which is used to encrypt the data (once the data owner submits the symmetrical key–encrypted data onto the IPFS). This second stage ensures that the data can be securely decrypted and re-encrypted using another pair of keys without access to the original data owner.

Furthermore, the data owner (seller) creates a smart contract that contains the hash of the mentioned components to act as the address of the data by minting the NFT as per the ERC-721 protocol. Once a sale is finalized (or a purchase action occurs), the data owner creates a re-encryption key from the public key of the data requester (buyer) and its own private key to send to the re-encryption oracle. This symmetrical key is then used by the re-encryption oracle and is shared with the buyer. Once the data are downloaded from the IPFS, the requester downloads the encrypted data, encrypted symmetrical keys, and the hash of the file. Subsequently, it decrypts the symmetrical key along with the data using its private key and decrypts the data again with that symmetrical key. The data requester (buyer) can then either choose to relist these data or use them for the analysis.

#### Reputation Models for Users and Data Sets

Reputation models enable buyers and sellers to evaluate each other and make informed decisions about transactions:

1. Rating and review systems: in this model, buyers and sellers can rate and review each other based on their experiences with the transaction. This allows other users to see the average rating and read reviews to help them gauge the reputation of a particular user. However, a weighting mechanism that weights subject matter experts, along with retail users (buyers or sellers), could prevent fraudulent reviews. Such a system will also need both manual and third-party moderation for the verification of content.
2. Verification systems: the marketplace can adopt verification systems to confirm the identity and credentials of users. This can help to build trust and may improve the reputation of verified users. An antimoney laundering or know-your-customer system that ties into the social security network or the credit profile can help validate real users or firms.
3. Feedback systems: feedback systems allow users to provide detailed feedback about their experiences with specific transactions. This feedback can be used to inform other users about the reputation of a particular user.
4. Trust networks: trust networks are systems that allow users to build relationships with each other and establish a reputation based on those relationships. Such trust networks within the context of a marketplace can enable supply chain kind of activities wherein buyers repeatedly trade with similar sellers, and sellers are able to preorder data sets to meet their analysis needs in the future.
5. Social media integration: marketplaces can integrate with social media platforms to allow users to connect their accounts and build their reputation based on their activity on those platforms. In the current design of a marketplace, we have integrated users with the social media platform.
6. Penalizing fraudulent data submitters: marketplaces can levy fines or completely blacklist users who engage in fraudulent data practices, such as offering fabricated data.

#### Data Fabrication Defense

A platform-level data-correctness strategy includes a combination of reputation mechanism design, statistical validation for data, onboarding validation for the data seller through third-party oracles, and penalization of the vendor upon detection of fraud by third-party vendors. In our design, we enabled the data description metadata entered by the user, which can be used to validate the data by third parties.

#### Pricing and Royalty Mechanism for Data

We created 2 smart contracts, one in which the value is transferred between the buyer and seller and another in which a proportion of the sale price at each transaction is transferred to the original creator (owner) of the data. This mechanism gives the data owner a market mechanism and an incentive to offer their data to the marketplace. Royalties to downstream and upstream sellers for personal data incentivize all players in the marketplace.

### Implementation

#### User Registration

We registered each user in the marketplace along with the user’s wallet ID and social media profiles to enable the user to list data. The data listed each time can be validated for fictitious or simulated data through a combination of third-party validation oracles and statistical analysis techniques to detect patterns of fraud. Figure 1 shows the user registration flow in the system. Multimedia Appendix 3 provides a video demonstration of the platform using Ethereum.

#### Premarket Validation

**Figure 6:**
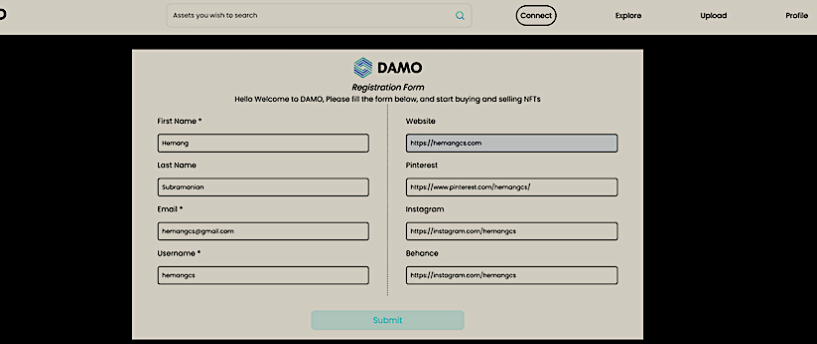
User preregistration with social media profile to check validity. NFT: nonfungible token.

When the PGHD data record is uploaded onto the IPFS, in the backend, a record on the blockchain will point to the unique CID on the IPFS. If the web service provider or marketplace wants to enable users to transact, the provider can pin the record onto a particular hosted node on the IPFS. Subramanian and Subramanian [31] described IPFS functionality, data storage, and use in the context of digital pathology. We used a similar mechanism for marketplace functionality and data storage, where metadata are stored, specifically pointing to the actual data on the IPFS. The CID pertaining to the metadata will reside in the blockchain record and is minted as an NFT (Figure 6).

**Figure 7:**
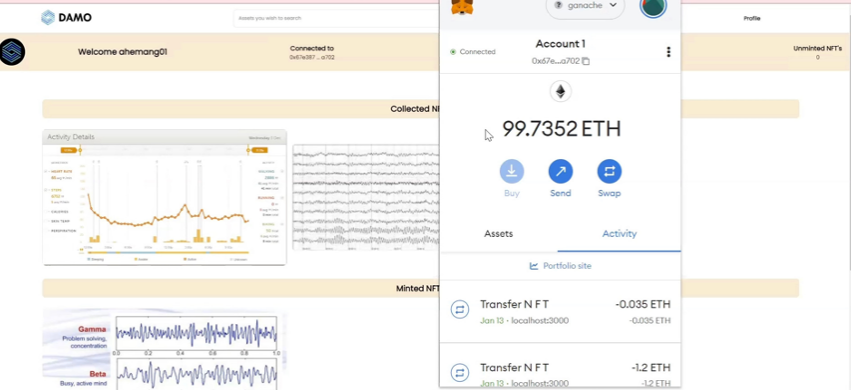
User flow depicting data upload and mint functionality. ETH: Ethereum; NFT: nonfungible token.

Figure 7 shows a screenshot of the user interface wherein users, upon logging into their wallets and identifying themselves, can see all the minted tokens. Each minted token is associated with an IPFS record that contains metadata pertaining to the uploaded data set. Furthermore, Figure 2 shows the interaction wherein the data are purchased using the wallet balance and the transfer of NFT. These metadata are listed in Listing 1 in Multimedia Appendix 1. In addition, when data are uploaded onto the IPFS, the tokens cannot be minted because of issues such as network connectivity, insufficient wallet balance, or high network traffic. The unminted tokens could later be minted by supplying sufficient balance to the user and later be used for listing on the decentralized marketplace.

**Figure 8:**
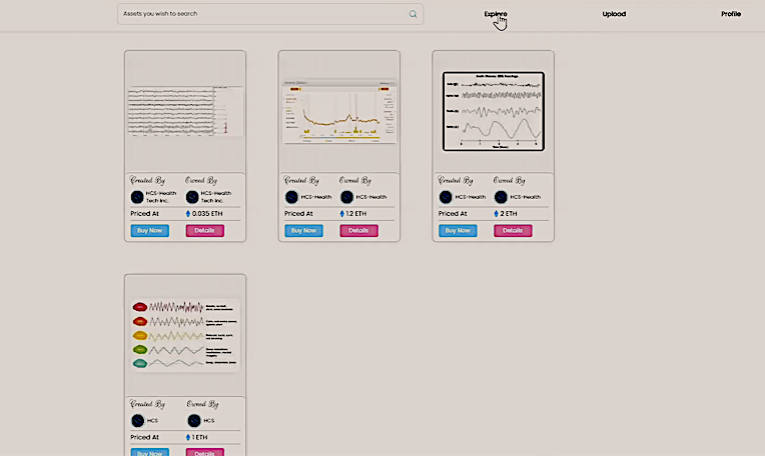
The user interface lists all these minted tokens on the network. Each user gets a separate listing, excluding their owned tokens available for sale in the marketplace.

Figure 8 shows the user interface of the decentralized marketplace displaying the listings. Although this user interface is implemented in HTML or Cascading Style Sheets, the web3 platform responsible for creating the listings platform can also supply a Representational State Transfer API for third parties to create and display listings.

### Data Categories

There are 3 categories of assets in the marketplace, unique to each wallet. The first category is “minted” NFTs that an owner can list in the marketplace for immediate transactional sale by a different user. Similarly, the second category is “collected NFTs,” which are just collections of digital health data attributed to the user but are not currently listed for sale. The third category of data accessible to the user not minted yet is listed as “unminted.” These records are not yet available on the blockchain for transactions. Code Listing 3 in Multimedia Appendix 1 lists the key functions used to create the listings. The JavaScript interfaces with the IPFS and the web3 smart contract and enables users to mint, list, and purchase tokens.

### HIPAA Support of the PGHD Marketplace

**Figure 9:**
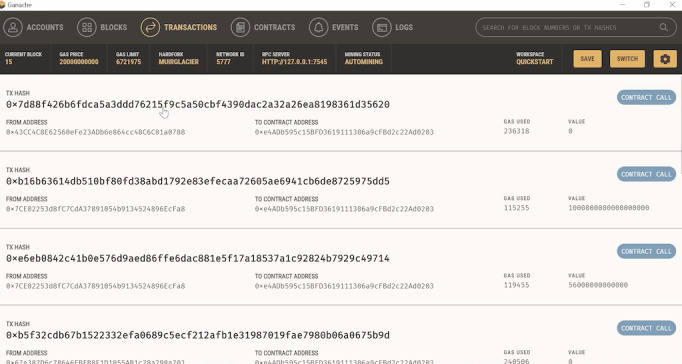
The contract address and transactions are done with respect to the nonfungible token creation.

HIPAA requires covered entities to protect individuals’ health records and other identifiable health information by requiring appropriate safeguards to protect privacy and by setting limits and conditions on the uses and disclosures that may be made of such information. Our design, in which personal device-generated data are uploaded into the IPFS, is encrypted and stored on the web. The blockchain provides a web-based transaction history of the data. For example, the minting of the aforementioned token is recorded on the blockchain and can be viewed on the Ethereum blockchain. The 6 aforementioned records that were minted with different Ethereum prices can be located by scanning the contract address on the network. We can examine which wallet transferred the newly created and minted NFT. In addition, each time the data are transferred, the original data owner earns a royalty, and the platform’s wallet also earns a share of the revenues. Figure 9 illustrates the creation of the contract and the set of transactions performed on the same.

In the subsequent section, we provide support for the various directives recommended by HIPAA. Figure S1 in Multimedia Appendix 1 displays the details of the transaction used to transfer the token from one address to another after paying the requisite fees. Note that the transaction uses the *TransferNFT* function, which transfers ownership from wallet A to wallet B (Figure S1 in Multimedia Appendix 1).

### HIPAA Regulations About Device-Generated Data

Our marketplace supports the following requirements with respect to PGHD as follows:

- Use a password or other user authentication: data owners access the marketplace with their private keys. Similarly, sellers, buyers, and value-added data service providers all access the marketplace using private keys.
- Install and enable encryption: all data can be encrypted as per the encryption design. The section on Health Data Encryption discusses how the data are encrypted and re-encrypted. At the storage level, users could choose to encrypt and send data using their public key (or a separate key), and their wallet software could enable access to these data later. Similarly, users could list a representative graphic at the marketplace level and encrypt the actual data for storage ion the IPFS.
- Install and activate remote wiping or disabling: as discussed, data can be deleted at the marketplace level by the owner using the burn functionality of the NFT, which removes the listing. However, the data can never be fully deleted from the IPFS, which replicates and stores data across nodes. Only data residing on pinned nodes can be deleted and submitted for garbage collection. Once the token is burned, the keys to decrypt the data are also deleted by the multiparty authentication, making these data inaccessible. The platform never sets an automatic burn for the token, rather the owner can invoke the burn by design.
- Install and enable a firewall: firewalls are installed by hosting providers or app providers if they choose to expose the marketplace via an app.
- Install and enable security software: the marketplace user interface operates via http secure and a web application. The security software and firewalls are installed on the server, which runs the user interface for the marketplace that interacts with the backend blockchain. In addition, third-party smart-contract security mechanisms help audit the blockchain's software contracts, enabling the Ethereum virtual machine to ensure compatibility (both forward and reverse).
- Keep the security software up to date: when updated, the blockchain software or IPFS will automatically update the required software. For example, the Ethereum blockchain moved to version 2 since the last submission, and the IPFS released its newest version. However, the marketplace user interfaces are controlled by those who enable a user interface, who are responsible for updating the software as per the new infrastructure protocols.
- Maintain physical control: access to data is controlled by the user who owns the private keys of the wallet, which uploaded the data onto the platform. The web API or marketplace user interface providers do not own or control it. Access monitoring is an additional responsibility, which the blockchain in itself does for all data that move through its system.
- Use adequate security to send or receive health information over public Wi-Fi networks.
- There are no data transferred to the public Wi-Fi networks, except when the record is uploaded onto the IPFS or downloaded by the buyer. It is encrypted whenever the data are uploaded and when they are downloaded or encrypted for later decryption. The encryption schema discusses the same.
- Delete all stored health information before discarding or reusing it: while the token can be delinked and delisted from the marketplace, actual data on the IPFS can never fully be deleted. Once the token is delisted, the re-encryption oracle will not enable anyone to re-encrypt the data, since the smart contract will not be able to detect the token (refer to the
  Health Data Encryption
  section). In addition, the MPA service can delete the encryption keys, thereby making it difficult to erase the data. Thus, although data continue to reside on the IPFS, it will not be accessible for decryption or later use.

## Results

We evaluated our prototype against the goals set out in the design phase:

- Goal 1: data access—this was fully implemented and demonstrated. Data access is permitted between different sellers and buyers, that is, buyers must have the right to access the data they purchased and sellers should have the ability to own and control the data and copies of data. Royalty is paid to the original creator of the data perennially on the marketplace, thus incentivizing data submission. Users can choose whether to list data that they own.
- Goal 2: marketplace workflow functionality—marketplace functionality for data was implemented. Sellers offer a fixed pricing mechanism and buyers can use their personal balances to debit currency to buy tokens.
- Goal 3: data encryption support—we implemented a partial version of the study by Battah et al [32] to support multiparty authentication for access of data. This supports data encryption to ensure that only the data owner can access the data, while other users cannot see the data.
- Goal 4: HIPAA compliance—we partially supported HIPAA features, as discussed. Certain limitations owing to limitations on the IPFS prevent full support of HIPAA functionality.
- Goal 5: reputation model—we presented various options to create a reputation model on the platform but implemented the user registration part that can be moderated. We collected all social media information to gauge user reliability on the platform and to validate users.

The marketplace addresses the key requirements and objectives that enable the monetization of health data in a fair and transparent manner. Similarly, it meets the goals set out to achieve. Next, we discuss the limitations of such a marketplace and future work.

## Discussion

### Governance

Decentralized marketplaces require governance structures that are not centrally controlled and managed. Governance structures provide oversight, management control, approvals for enhancements to the platform, reward mechanisms, and a formal structure answerable to the law of the land. A consortium-based approach is recommended wherein representatives of health data providers, buyers, and value-added service providers participate in a voting-based decision-making system. Penalizing collusion can be a deterrent to any attempt to thwart decentralized governance. In a consortium-based governance approach, all stakeholders, including the legal community, public, buyers, and sellers, have a stake in the platform’s decision-making process. Another approach is that of a decentralized autonomous organization, where governance tokens (using smart contracts) could be issued to users participating in the platform’s governance. Although HS has prototyped a token-based governance model for such a marketplace, the complexities in defining briefly such a schema can be the subject of future research.

### Limitations and Future Research

First, the creation of such a marketplace, while allowing the acceleration of data provision in markets, can increase the quantity of data available in marketplaces.

However, excessive data listed in the marketplace can increase the search costs for end users unless the marketplace creator implements a local search. Second, owing to the use of blockchain, the IPFS, and other technologies, where users can upload and store data inexpensively, it is likely that many users could start using such a platform as a data storage device. To solve these issues, platform operators should design and operate recommendation systems that work in tandem with users uploading and trading data, providing ratings and reviews for both data sets and data providers.

Third, the onboarding of data providers should be controlled by firms operating the platform rather than a free-for-all service, where people can use it for various nefarious purposes. This provides additional monetization opportunities for marketplace creators, data providers, or device manufacturers. “Unminted” data and unlisted data could be reduced to eliminate free renting. Fourth, owing to the decentralized nature of such marketplaces, it is important to realize that decentralization also leads to challenges with account integrity owing to the anonymity provided by the blockchain. Fifth, decentralized marketplaces pose a threat to existing industry structures, where major hardware creators such as Apple and Fitbit (Google Inc) dominate wearables. As a result, conflicts with the survival of such a marketplace could be exacerbated. Sixth, the legal and regulatory implications for a marketplace that trades in PGHD while generating secondary value-added services (such as diagnostic ability) have not yet been investigated in this paper and could be the subject of future research. Seventh, the scalability of the solution when data size exceed petabytes needs to be investigated with layer 2 solutions, such as Filecoin, ArWeave, and Storj. Future research can highlight more performant solutions based on the IPFS.

### Conclusions

In this paper, we proposed, designed, and prototyped a decentralized marketplace for PGHD data. We proposed a mechanism by which different participants, such as data creators, sellers, and value-added service providers, can monetize data transparently. Similarly, our design attempts to support the HIPAA regulations that provide privacy, security, and legal protection to users, platform creators, and other stakeholders in the ecosystem.

Such a marketplace can improve (1) the quality of data available in the marketplace and will ensure that (2) more high-quality data are available for artificial intelligence–driven analysis and diagnosis of diseases. The salient features of our decentralized PGHD marketplace are as follows:

- In a health care data marketplace, not only do data providers such as patients, data aggregators, or data enablers benefit in deriving value for the data they create and own, but also intermediaries such as diagnostics laboratories, data aggregators, and application creators are benefited.
- The data provenance issue is mitigated because the blockchain supports traceability and historical audition of ownership changes and data origins.
- In the PGHD marketplace, the architecture facilitates different buyers and sellers to offer their data in the market, thereby keeping the markets more efficient in terms of access, price, and security.
- In the PGHD marketplace, data owners and those who submit data have the ability to earn revenue in the marketplace and royalties for the data they provide, thus monetizing their own health data.

## Appendix

Multimedia Appendix 1 Important code snippet listings, metadata listings, and blockchain information that is directly sourced from the public blockchain infrastructure.

Multimedia Appendix 2 Tables defining the differences between decentralized marketplaces and other forms of health exchanges, including best practices for key management.

Multimedia Appendix 3 Demonstration of the decentralized marketplace.

## Abbreviations

API: application programming interface
CID: content identifier
ERC: Ethereum Request for Comments
HIE: health information exchange
HIPAA: Health Insurance Portability and Accountability Act
IPFS: interplanetary file system
NFT: nonfungible token
PGHD: patient-generated health data
RPDR: research patient data repository
