# Supplementary material for: A Decentralized Marketplace for Patient-Generated Health Data: Design Science Approach
Source: J Med Internet Res. 2023 Feb 27;25:e42743. doi: 10.2196/42743 (PMC10012005; doi:10.2196/42743)
Supplement: Multimedia Appendix 1 [file jmir_v25i1e42743_app1.docx]

Appendix A. MultiMedia Appendix

Code Listing 1. JSON metadata file that stores the parameters of the meta Data

*{*

*"name": "NFT for BP monitor for a year",*

*"description": "This dataset consists of Blood pressure data for owner for a year.",*

*"data file": https://ipfs.io/ipfs/QmT5NvUtoM5nWFfrQdVrFtvGfKFmG7AHE8P34isapyhCxX/BP.xlsx.*

*“Resolution” : 10000000 dpi*

*"device": "3DHISTECH Panoramic"*

*"Clinic": "Monta vista pharmaceuticals and diagnostics ltd. Ohio, USA"*

*"Physician ID": 198765482*

*"Owner": Patient Name*

*"Owner wallet ID": Patient ID*

*}*

Code Listing 2. Smart contract code snipped that implments creation of the token, minting functionality and transferring the NFT, setting a price for the NFT.

|  |
| --- |

contract DAMO is ERC721, ERC165, ERC721Metadata, ERC721Enumerable, ERC2981 {

//Metadata

string private _tokenName;

string private _tokenSymbol;

uint16 private id = 1;

address public contract_owner;

//enumeration

uint256[] private allTokens;

mapping(address => mapping(uint256 => uint256)) private _ownedTokens;

mapping(uint256 => uint256) private _ownedTokensIndex;

function tokenURI(uint256 _tokenId)

public

view

virtual

override

returns (string memory)

{

require(_exists(_tokenId), "Token does not exist!!");

return _tokenURIs[_tokenId];

}

function balanceOf(address _owner)

public

view

virtual

override

returns (uint256)

{

require(_owner != address(0), "Invalid Query");

return _balance[_owner];

}

function ownerOf(uint256 _tokenId)

public

view

virtual

override

returns (address)

{

address owner = _owners[_tokenId];

require(owner != address(0), "Invalid NFT/Token");

return owner;

}

function safeTransferFrom(

address _from,

address _to,

uint256 _tokenId

) public payable virtual override {

require(_to != address(0), "Invalid Reciever Address");

require(_from != address(0), "Invalid sender address");

require(_owners[_tokenId] == _from, "Not the owner");

require(_exists(_tokenId), "Invalid NFT/Token");

safeTransferFrom(_from, _to, _tokenId, "");

}

function _transfer(

address _from,

address _to,

uint256 _tokenId

) internal virtual {

require(_exists(_tokenId), "NFT does not exist");

require(balanceOf(_from) > 0, "Sender doesn't own any tokens");

require(

msg.value >= tokenToPrice[_tokenId],

"Pay the right price for token"

);

_beforeTokenTransfers(_to, _from, _tokenId);

_balance[_from] -= 1;

_balance[_to] += 1;

_owners[_tokenId] = _to;

uint256 royaltyAmount;

address receiver;

(receiver, royaltyAmount) = royaltyInfo(

_tokenId,

tokenToPrice[_tokenId]

);

payable(receiver).transfer(royaltyAmount / 2);

payable(contract_owner).transfer(royaltyAmount / 2);

payable(_from).transfer(msg.value - royaltyAmount);

tokenToPrice[_tokenId] = msg.value;

emit Transfer(_from, _to, _tokenId);

}

function changePrice(uint256 price, uint256 tokenId) public virtual {

require(_exists(tokenId), "Token does not exists");

require(msg.sender == _owners[tokenId], "You are not the owner");

tokenToPrice[tokenId] = price;

}

function mintToken(

address to,

string memory uri,

uint256 price,

address creator

) public payable {

tokenToPrice[id] = price;

royaltyReceiver[id] = creator;

mint(to, uri);

}

function mint(address to, string memory uri) public payable {

_beforeTokenTransfers(to, address(0), id);

_owners[id] = to;

_balance[to] += 1;

_setTokenURI(id, uri);

emit Transfer(address(0), to, id);

id++;

}

function safeTransferFrom(

address _from,

address _to,

uint256 _tokenId,

bytes memory data

) public payable virtual override {

require(_to != address(0), "Invalid Reciever Address");

// require(_from == msg.sender, "Not the current owner");

require(_exists(_tokenId), "Invalid NFT/Token");

_safeTransfer(_from, _to, _tokenId, data);

}

function _safeTransfer(

address from,

address to,

uint256 tokenId,

bytes memory _data

) internal virtual {

_transfer(from, to, tokenId);

require(

_checkOnERC721Received(from, to, tokenId, _data),

"ERC721: transfer to non ERC721Receiver implementer"

);

}

function transferFrom(

address _from,

address _to,

uint256 _tokenId

) public payable virtual override {

require(_exists(_tokenId), "Token does not exist!!");

require(_owners[_tokenId] == msg.sender, "You are not the owner");

require(_from == _owners[_tokenId], "You are not the owner!");

require(_to != address(0), "Invalid address");

_transfer(_from, _to, _tokenId);

}

function transferNFT(

address _from,

address _to,

uint256 tokenId,

uint256 price

) public payable {

safeTransferFrom(_from, _to, tokenId);

changePrice(price, tokenId);

}

Code Listing 3. Web User Interface that displays the User Interface for users to register with their wallets, mint NFTs and send data over to others.

|  |
| --- |

let web3;

let web3connection = {

account: null,

web3: null,

};

//Opening popup

function openModal(modal) {

if (modal == null) return;

modal.classList.add("active");

overlay.classList.add("active");

}

//Closing popup

function closeModal(modal) {

if (modal == null) return;

modal.classList.remove("active");

overlay.classList.remove("active");

}

if (photo) {

photo.addEventListener("change", async (event) => {

extension = photo.files[0].name.split(".").slice(-1)[0];

const fileName = photo.files[0].name.split(".")[0];

if (fileName.length > 22) {

photoLabel.textContent = fileName.slice(0, 21) + "...." + extension;

} else {

photoLabel.textContent = fileName + "." + extension;

}

// Removing browse button and making upload button to appear

browse.classList.remove("active");

imageSubmitBtn.classList.add("active");

imageSubmitBtn.textContent = "Upload";

imageSubmitBtn.disabled = false;

const reader = new FileReader();

reader.addEventListener("load", async function () {

photoPreview.style.display = "block";

photoPreview.innerHTML = '<img src="' + this.result + '" />';

});

reader.readAsDataURL(photo.files[0]);

});

}

const priceEther = document.getElementById("price-ether");

const creator = document.getElementById("creator");

const sameAsOwnerCheckbox = document.querySelector(".same-as-owner");

sameAsOwnerCheckbox.addEventListener("change", (e) => {

if (e.target.checked) {

creator.value = web3connection.account;

creator.disabled = true;

} else {

creator.value = "";

creator.disabled = false;

}

});

const checkValidAddress = (address) => {

if (address.startsWith("0x") && address.length === 42) {

return true;

} else return false;

};

// Submit event in image upload form

if (imageSubmitBtn) {

imageSubmitBtn.addEventListener("click", async (event) => {

if (priceEther.value == "" || creator.value == "") {

showAlert("Please fill all the fields", "failure");

return;

}

if (!checkValidAddress(creator.value)) {

showAlert("Please enter a valid creator address", "failure");

return;

}

event.preventDefault();

imageSubmitBtn.textContent = "Uploading...";

imageSubmitBtn.disabled = true;

const price = web3.utils.toWei(`${priceEther.value}`, "ether");

const formData = new FormData(form);

try {

const res = await axios.post(

"http://localhost:3000/api/v1/image/ipfsUpload",

formData

);

if (res.data.status === "success") {

CID = res.data.data.cid;

const account = web3connection.account;

try {

const payload = {

CID,

account: account,

price,

creator: creator.value,

previewPath: `http://localhost:3000/Images/previews/${CID}.${

extension === "gif" || extension === "webp" ? "webp" : "jpeg"

}`,

};

const res1 = await axios.post(

"http://localhost:3000/api/v1/image/upload",

payload

);

if (res1.data.status === "success") {

showAlert(

`Uploaded Successfully...<br>Account:<br>${account.slice(

0,

21

)}<br>${account.slice(21)}`,

"success"

);

showAlert(

`Mint Your Token.<br>Your CID:<br>${res.data.data.cid.slice(

0,

23

)}<br>${res.data.data.cid.slice(23)}`,

"success"

);

imageSubmitBtn.classList.remove("active");

priceEther.disabled = true;

creator.disabled = true;

mintBtn.classList.add("active");

mintBtn.disabled = false;

if (mintBtn) {

mintBtn.dataset.cid = CID;

mintBtn.dataset.extension = extension;

}

} else {

browse.classList.add("active");

browse.textContent = "Try Again";

imageSubmitBtn.classList.remove("active");

mintBtn.classList.remove("active");

browse.disabled = false;

imageSubmitBtn.disabled = true;

mintBtn.disabled = true;

showAlert(`${res1.data.message}`, "failure");

}

} catch (err) {

console.log(err);

console.log("DB issue");

}

} else if (res.data.status === "failure") {

showAlert(

"Some error has occured while uploading into ipfs... <br>Try again by uploading the image again...",

"failure"

);

imageSubmitBtn.textContent = "Try Again";

console.log(res);

setTimeout(() => {

closeModal(uploadModal);

}, 1000);

} else {

showAlert("Please, Upload Again...", "failure");

imageSubmitBtn.textContent = "Try Again";

setTimeout(() => {

closeModal(uploadModal);

}, 1000);

}

} catch (err) {

showAlert(

"Some error has occured while uploading into ipfs... <br>Try again by uploading the image again...",

"failure"

);

imageSubmitBtn.textContent = "Try Again";

console.log(err);

}

});

}

Figure 1. The sequence of transactions which are used per wallet.

| 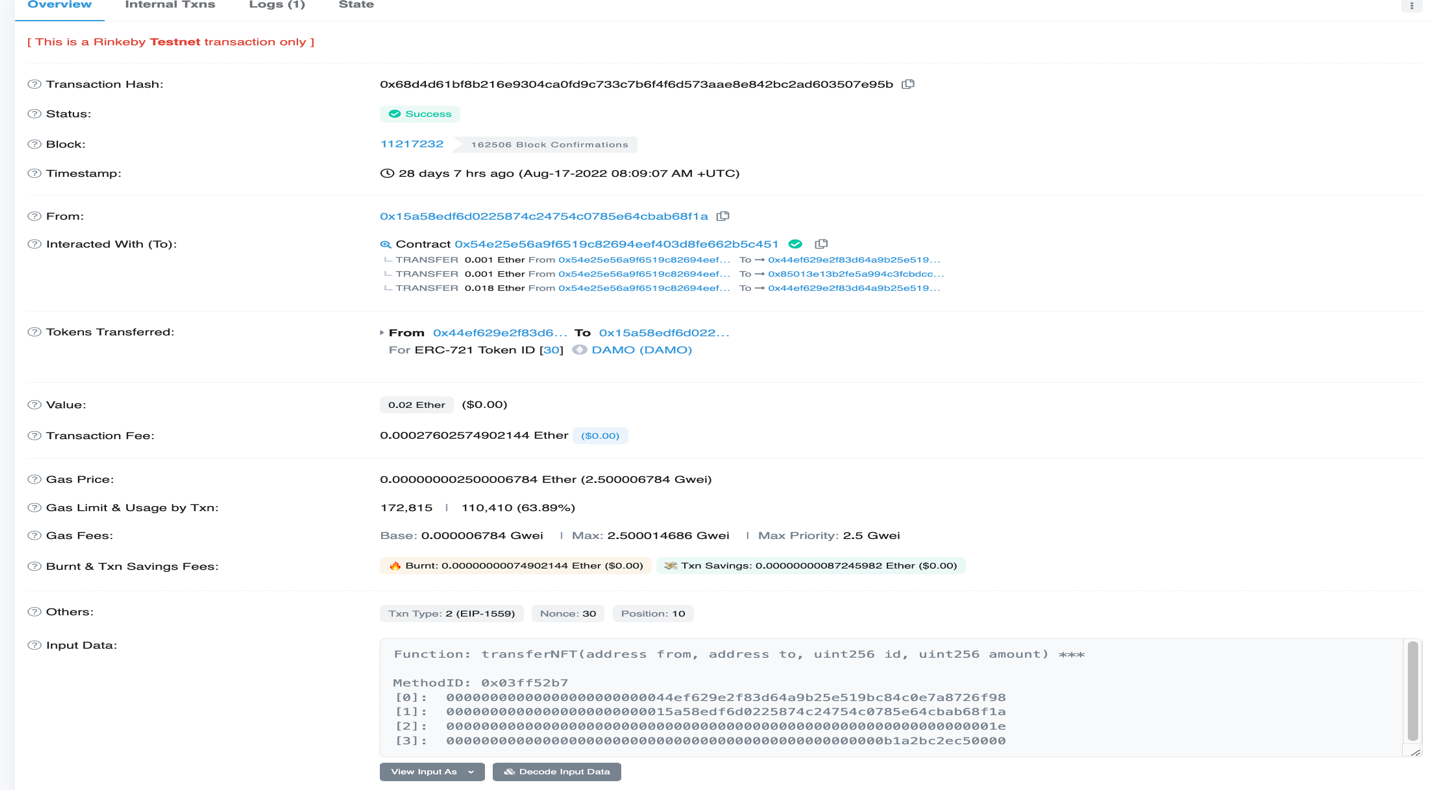 |
| --- |
