# Supplementary material for: A Decentralized Marketplace for Patient-Generated Health Data: Design Science Approach
Source: J Med Internet Res. 2023 Feb 27;25:e42743. doi: 10.2196/42743 (PMC10012005; doi:10.2196/42743)
Supplement: Multimedia Appendix 2 [file jmir_v25i1e42743_app2.docx]

# Appendix B:

 Table S1. Illustration of differences between centralized health data stores and Decentralized PGHD data marketplaces targeted in our design

| Features | Definition | Centralized data systems such as HIEs, RDPRs . | Decentralized marketplace. |
| --- | --- | --- | --- |
| Access Control | The ability to manage who controls the data upon creation of PGHD data. The ability to manage who owns the data upon creation of PGHD data. | Database administrators who manage access can access data on RDPR, HIE, etc. already have access control. Most health exchanges, RDPRs, and HIE, while allowing the controlled exchange of information between organizations, do not allow individuals to offer their own PGHD data. | Decentralized access to data is based on those who possess the keys to view data. Access is regulated as seen in this; where only data owners can access the data. |
| Access Monitoring | The ability to monitor who currently has access to which record at what timeframe. | Data access must be separately documented, and such documentation must be preserved separately. While access and monitoring in centralized systems happen via additional software that generates audit trails and audit logs. | Any transaction is recorded on the blockchain by design and systems can be queried to obtain the original transaction log. |
| Data Security | The ability to prevent unwanted leaks, modifications, and creatives for data. | As secure as the data warehouse and/or as secure as the ability to login into the network.There are many instances in the recent past where centralized health data warehouses are subject to all kinds of security attacks, and ransomware. | Data security has two components on the blockchain.  The marketplace ensures that data submitted onto the platform is accessible only to the owner, encryption ensures that data is private. Transactional anonymization is also possible by zk-proofed transactions and homo-morphic encryption  While the platform is responsible for securing the data, users are responsible for their own keys. Refer Appendix A for best practices about key security. |
| Data Privacy Enforcement | Data is as private as the laws governing the data repository. | Users have no say in such matters. Firms controlling the data ensure that data is private, and only certain users have access. | Designers of the decentralized marketplace functions are responsible for data privacy. |
| Data Validity | The ability to validate the data with respect to its content, time of creation, storage, etc. | It will be difficult for end users to verify data validity. Users will have to trust the centralized administrators of the data. | Data will never be manipulatable on the system since decentralized storage applications. |
| Data Verifiability | The ability to verify different data. e.g, past access, modifications, transmission. | Once data is modified within the Datawarehouse, a user would have to access the data logs to validate the transaction. Data can be easily modified, i.e., overwritten, appended, or modified with database queries. Data fields could be moved to previous states through rollbacks, etc. | Data can never change on the blockchain since the data once stored on the blockchain is consistent forever. Even if the data changes, such changes will forever consistently perennially be recorded in a new block. |
| Data traceability | Ability to trace data - the change of data ownership, the change of access, removal of data. | Each time the data moves on the network, it creates a separate data entry. Recent investments in audit technology has provided such features out of the box, but there are no standards available except those implemented by the system. | The ability to trace the movement of health data from its creation to its current user is unique to the blockchain, which retains the records of the chains of data. |
| Data Provenance | Sourcing the origins of data and recording this origin for future verification. Provenance provides certification of originality for the dataset. | Since provenance is also controlled by the central platform, we can only trust what the central platform provides. | The ability to trace data and validate the origins of data from storage and records prevalent therein. |
| Data Sufficiency for Machine Learning | The ability to verify whether the data has variety, is large and can be used to train models. | The ability to sustain and retain a sufficiently large dataset to make Machine Learning (ML) models generate accurate data results. | The ability to create a sufficiently large dataset with Machine Language (ML) models to generate accurate results. |
| Data Variety for Machine Learning | The ability to source a variety of data for applying machine learning techniques. | The centralized data store will work if the centralized entities can physically solicit data variety from their users. | Since multiple users can buy data across the system, it is worthwhile trying to get multiple users to create a good dataset. |
| Value Added Services | The ability to provide and offer value-added services such as data visualization, analytics, data cleaning, and data mining services | This depends on the ability of the central hosting platform to hire/recruit and manage such data analytics offerings | Multiple vendors and data analytics businesses can engage with the platform to facilitate such business offerings. |

2.0 Comparison between Decentralized marketplaces, Health Information Exchanges and Integrated Data Repositories.

Table S2 . Comparing Decentralized marketplaces with HIEs, IDPRS

|  | Decentralized Health Data Marketplace | Health Information Exchanges | Integrated Data Repositories |
| --- | --- | --- | --- |
| Storage and data types | It can be used for data which can be self-serve where users control modifications of data. Marketplace creators can create and manage their storage with such a framework. | Usually used amongst different hospital/clinical settings for clinical data. Controlled by the institution that usually incurs the cost of storage – usually on the cloud. | Used for Clinical data and among large hospital systems |
| Data Quality | Competition among users can improve data quality | Very difficult to ascertain since different players participate in the market | Supported by the researchers and others involved in the ecosystem. |
| Data Privacy | Privacy is ensured since the data owner and mhealth and uhealth data providers can encrypt and store their data. Only they have access. | Privacy as much as is provided by HIE provider and database. Data ownership is not entirely enforced, and the exchange controls. | Privacy as much as Integrated Data Repositories enable . |
| Data Characteristics |  |  |  |
| User Monetization | Enables data providers and creators to monetize the data so created. This provides a mechanism for market competition, which will improve the quality and quantity of data over time. | HIEs enable the exchange of information, and rarely do users, or data providers enable the monetization of data | This a pure research data. While data creators and owners don't monetize data, private enterprises accessing data can make and analyze data and later monetize it. |
| Security | The blockchain is extremely secure and cannot be hacked. Users control and maintain their keys to access data. However, even if a user loses his key, his records will become inaccessible rather than lost. | The single point of failure introduced by the centralized HIE. Recent data has indicated that several centralized healthcare systems are hacked, and patient records are stolen. | Very complex to manage and maintain due to the disparity in data formats, databases, and security frameworks. Amenable to easy hacks . |
| Marketplace functionality | This is a marketplace by design. | Data ownership, digital data rights, etc., are difficult to track with respect to uhealth data and mhealth data. No marketplace functionality is inbuilt. However, HIEs can choose to create marketplaces when sufficient data is available. | No marketplace functionality is possible since this is for pure research purposes. As a result, data cleaning, scrubbing processes, etc., are extremely difficult to retain and control. |

We believe that the rate of innovation in applying AI/telemedicine/automated diagnosis can accelerate if data is made available in a controlled, auditable, and non-user identifiable way while providing financial incentives to those that create, own, and trade in such data. A marketplace approach to handling user data not only ensures that such an evolution in healthcare is plausible. Such an approach can ensure that the different sides of the marketplace are adequately incentivized to create high-quality data. The incentive mechanisms of these marketplaces not only make data accurate and secure but also adhere to the laws governing the privacy, security, and auditability of healthcare data.

3.0 Key management principles and best practices to not loose keys:

There are a few key ways to securely store the keys of your crypto-wallet:

1.     Use a hardware wallet: A hardware wallet is a physical device that stores your private keys offline. This makes it much more difficult for your keys to be stolen or compromised, as the device is not connected to the internet. Hardware wallets are considered to be one of the most secure options for storing crypto-currency keys.

2.     Use a paper wallet: A paper wallet is a physical copy of your private keys that is printed on paper. This can be a secure way to store your keys, as long as you keep the paper in a safe place and make sure it is not lost or damaged.

3.     Use a software wallet with strong security measures: Software wallets, which are stored on your computer or mobile device, can also be a secure option if they have strong security measures in place. Look for a wallet that offers features such as two-factor authentication and multi-sig support.

It's important to remember that no matter which storage method you choose, it's crucial to keep your keys safe and secure. This may include keeping them in a secure location, using strong passwords, and regularly backing up your keys.

3.0 IPFS file permanent file deletion

To delete a file from IPFS, you can use the ipfs pin rm command followed by the hash of the file you want to delete. This will remove the pin on the file, which allows it to be garbage collected by IPFS.

ipfs pin rm <hash>

Keep in mind that deleting a file from IPFS does not remove copies of the file that may exist on other nodes in the network. Once a file is added to IPFS, it can be replicated and shared across the network, so it is not possible to completely delete a file from IPFS.

If you want to remove a file from your local IPFS node, you can use the ipfs repo gc command, which will remove all objects that are not being used by pinned objects from your local repository. This will not remove the file from the IPFS network, but it will remove it from your local node.

ipfs repo gc

It is important to note that, Encrypted data even if it exists on IPFS will not be accessible to the end user once the NFT token is burnt in the marketplace, since the encryption oracle and MPA wallet will also destroy its encryption keys.

4.0 Alternates to IPFS for data protection.

While on the overall, the burden for data protection is placed on encryption, future versions of the marketplace could consider variants of IPFS storage such as FileCoin, Sia, Arweave, Storj and MaidSafe. Off these technologies and variants, FileCoin is a layer built atop the IPFS, and provides a custom-encryption-decryption scheme to make this data private. In the Filecoin network, data is encrypted and divided into small chunks, called "ciphertexts," before it is stored on the network. Each ciphertext is encrypted using a unique key, which is itself encrypted and stored along with the ciphertext. This key is known as the "ciphertext key." When a client wants to store data on the Filecoin network, they first generate a unique key for the data, called the "data key." The client then uses this key to encrypt the data, producing the ciphertexts. The client then sends the ciphertexts and the encrypted data keys to the network, where they are stored on a distributed set of storage miners.

To retrieve the data, the client must provide the correct data key, which is used to decrypt the ciphertext key, which is in turn used to decrypt the ciphertexts and reconstruct the original data. This process ensures that the data is secure while it is stored on the network, and can only be accessed by someone who has the correct data key.
